# Protective Effects of *Gynostemma pentaphyllum* (var. Ginpent) against Lipopolysaccharide-Induced Inflammation and Motor Alteration in Mice

**DOI:** 10.3390/molecules26030570

**Published:** 2021-01-22

**Authors:** Andrea Mastinu, Sara Anna Bonini, Marika Premoli, Giuseppina Maccarinelli, Eileen Mac Sweeney, Leilei Zhang, Luigi Lucini, Maurizio Memo

**Affiliations:** 1 Department of Molecular and Translational Medicine, Division of Pharmacology, University of Brescia, 25123 Brescia, Italy; sara.bonini@unibs.it (S.A.B.); m.premoli002@unibs.it (M.P.); giuseppina.maccarinelli@unibs.it (G.M.); e.macsweeney@studenti.unibs.it (E.M.S.); maurizio.memo@unibs.it (M.M.); 2 Department for Sustainable Food Process, Università Cattolica del Sacro Cuore, 29122 Piacenza, Italy; leilei.zhang@unicatt.it (L.Z.); luigi.lucini@unicatt.it (L.L.)

**Keywords:** *Gynostemma pentaphyllum*, supplement, anti-inflammatory, motility, ELISA

## Abstract

*Gynostemma pentaphyllum* (var. Ginpent) (GP) is a variety of Cucurbit with anti-inflammatory and antioxidant effects in patients. In this manuscript, the main components present in the dry extract of GP have been identified using Ultra High Performance Liquid Chromatography quadrupole-time-of-flight mass spectrometry (UHPLC/Q-TOF-MS). In addition, the anti-inflammatory action of GP was evaluated in animal models with acute peripheral inflammation and motor alteration induced by lipopolysaccharide. The results showed that GP dry extract is rich in secondary metabolites with potential antioxidant and anti-inflammatory properties. We found that the treatment with GP induced a recovery of motor function measured with the rotarod test and pole test, and a reduction in inflammatory cytokines such as interleukin-1β and interleukin-6 measured with the ELISA test. The data collected in this study on the effects of GP in in vivo models may help integrate the therapeutic strategies of inflammatory-based disorders.

## 1. Introduction

### 1.1. The Plant phytocomplex

Plants produce secondary metabolites in the leaves, stem or roots, with allelopathic, defensive or attractive functions towards other plants or animals [1,2,3]. Over the past 70 years, single secondary metabolites have been isolated, purified, and synthesized ex-novo and their biological activity has been evaluated [4,5,6,7]. Important therapeutic applications have been found for many of these molecules, but in other cases, clear and definitive results have not been obtained [8,9]. In recent decades, studies on the biological activity of phytocomplexes have been growing. The characterization of the phytocomplex does not focus on a single compound produced by the plant, but on the heterogeneous set of different molecules present in a specific area of the plant at a particular time of vegetative life [10,11]. This new approach allows the evaluation of many ecological parameters such as soil nutrients, abiotic and biotic stress, and the presence of other plants. These parameters affect the quantity and quality of the secondary metabolites present in the plant and consequently their biological activity [12,13,14]. Indeed, the ethnopharmacological properties of a phytocomplex do not always coincide with the biological effects observed for each single molecule present in it [15,16,17]. Starting from these considerations, the anti-inflammatory properties of the phytocomplex of a variety of *Gynostemma pentaphyllum* (var. Ginpent) (GP) have been studied.

### 1.2. Overview on Gynostemma pentaphyllum

GP belongs to the Cucurbitaceae, it is a perennial plant with a lianose habit, and it grows spontaneously in Indochina [18,19]. The studies present in the scientific literature exclusively concern the variety *Gynostemma pentaphyllum* (Thunb.) Makino (GTM) in several models [20,21,22,23]. Furthermore, the uses of GTM in traditional medicine are diverse, including the treatment of bronchitis, asthma, liver disorders, fat tissue disorders, and inflammatory and oncological pathologies [21,23,24,25]. GTM could reduce inflammatory heart damage by inhibiting the activation of NF-*κ*B p65 via the mitogen-activated protein kinase (MAPK) signalling pathway in the H9c2 cell model [26]. Furthermore, it has been reported that GTM extracts are involved in the inhibition of inflammatory signalling mediated by IL-6, IL-1β, COX-2, TNF-α and NO in RAW264.7 macrophage cells stimulated with lipopolysaccharide (LPS) [20]. Finally, GTM could inhibit the activation of the proinflammatory signalling pathways NF-*κ*B and STAT3 and the production of proinflammatory cytokines in intestinal inflammation models [23]. Although the anti-inflammatory action of GTM has been defined in different in vitro and in vivo studies, no model explores the effects related to motor behaviour. Furthermore, in vivo experimental results on the action of GP have not yet been performed.

In this work, the anti-inflammatory and motor coordination effects of a supplement based on GP were evaluated in mice treated with lipopolysaccharide (LPS). In vivo acute treatment with lipopolysaccharide is a standard method to induce inflammation, with the release of inflammatory cytokines and the alteration of some physiological functions, such as motor activity [27]. GP, a formulation already on the market as a food supplement (GinPent^®^, Leno, Italy) with potential adaptogenic, antidiabetic and lipid-lowering activities, was used for preventive purposes three days before the inflammatory stimulation. Furthermore, the effects of GP on motor coordination were evaluated with the pole and the rotarod test while the anti-inflammatory action was evaluated with the enzyme-linked immunosorbent assay (ELISA) test. Finally, the identification of some secondary metabolites present in the GP extract was performed.

## 2. Materials and Methods

### 2.1. Plant Material

**Figure 1:**
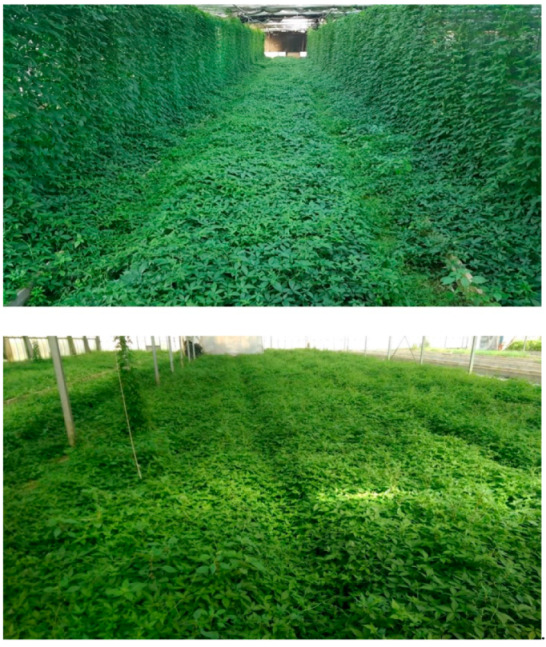
Crops of *Gynostemma pentaphyllum* (var. Ginpent) from Leno, Brescia, Italy.

GP is derived from a selected variety of the *Gynostemma pentaphyllum* (var. Ginpent) (Plant Reg. 8488) owned by AMBRAFARM srl, Leno, Italy (Figure 1). The whole plant of GP was collected from Leno (45°21′52.3″ N 10°11′53.9″ E), Province of Brescia, Italy, in October 2019. The leaves of GP were dried, chopped and pulverized before the mice treatment and the analytical procedures. One sample of GP was kept at the Department of Molecular and Translational Medicine, Division of Pharmacology, University of Brescia.

### 2.2. UHPLC-QTOF Analysis of Bioactive Compounds

In this work, triplicate specimens of the test sample were analysed by Ultra High Performance Liquid Chromatography quadrupole-time-of-flight mass spectrometry (UHPLC-ESI/QTOF-MS). To this aim, samples were extracted through Ultra-Turrax (VWR, Milano, Italy) as previously reported [28]. Thereafter, a 34 min linear elution gradient using a binary mixture of acetonitrile in water (from 5 to 94%, flow 220 µL/min, T = 35 °C) and a Zorbax Eclipse-plus column (75 × 2.1 mm i.d., 1.8 μm-Agilent Technologies, Santa Clara, CA, USA) were used for chromatographic separation [29]. The mass spectrometer was operated in full-scan mode and positive polarity, acquiring in the range 100–1200 *m*/*z* [28].

Raw data underwent mass and retention time alignment and were then deconvoluted in Agilent Profinder B.06 (Agilent, Santa Clara, CA, USA). Features were finally annotated according to a level 2 of confidence in annotation [30] against two databases, namely Phenol-Explorer 3.6 [31] and a custom database prepared from known saponins and phytosterols reported in literature.

Phenolic compounds were finally classified in classes according to Phenol-Explorer 3.6 annotations, and cumulate compound abundance from each class was quantified using calibration curves built from pure reference standards [32]. In more detail, catechin was used for flavanols, cyanidin for anthocyanins, luteolin for flavones and other remaining flavonoids, sesamin was used for lignans, ferulic acid for hydroxycinnamic acids and other phenolic acids, resveratrol for stilbenes, 5-pentadecylresorcinol alkylphenols, and tyrosol was used for tyrosols and other low molecular weight phenolics. Regarding saponins and phytosterols compound classes, they were quantified using Ginsenoside-Rb and cholesterol (Sigma-Aldrich, Milan, Italy) standard curves, respectively.

### 2.3. Animals and Treatment

All experiments were performed according to the European Union guidelines (CEE N°86/609) for the care and use of experimental animals and approved by the Italian Ministry of Health (381/2019-PR), Animal Care and Use Committee of the University of Brescia.

Forty male mice (2–3 months old, 25–30 g, B6;129PF2) fed a standard diet, and housed in 4-individuals/cage at controlled temperature and humidity, were used. Animals were fed ad libitum with a normal chow (D12450B, 10% fat, 70% carbohydrate, 20% protein, total 3.85 kcal/g; Research Diets Inc., New Brunswick). We randomly divided the animals into two groups balanced on body weight, with 20 mice per group. The groups were: control group treated with vehicle (VH, for three days, saline solution), group treated with GP (GP, for three days). The GP solution was prepared fresh every morning, starting from a pool of 10 capsules. The desired quantity was weighed, solubilized in physiological solution (NaCl 0.9%), sonicated, and administered by oral gavage (20 mg/kg).

**Figure 2:**
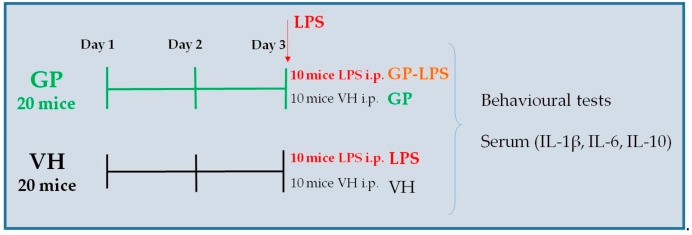
Experimental design applied to test the motility and anti-inflammatory effects of *Gynostemma pentaphyllum* (var. Ginpent) (GP). The groups were: control group treated with vehicle (VH, saline solution), group treated with Ginpent^®^ (GP). On the third day, ten GP (GP-LPS) and ten VH mice received the inflammatory stimulus, lipopolysaccharide (LPS).

On the third day, ten GP and ten VH mice received the inflammatory stimulus (lipopolysaccharide, LPS, L-3129 serotype 0127:B8, MilliporeSigma, Merck KGaA, Darmstadt, Germany). LPS was administered intraperitoneally (i.p.) at a dosage of 7.5 mg/kg. The dosage was chosen on the basis of data previously reported in the literature [33]. The remaining 10 GP animals and 10 VH animals received i.p. saline (Figure 2).

After about 6 h from the administration of the LPS, the animals were subjected to motor tests: “Pole Test” and “Rotarod test”.

### 2.4. Behavioural Test: Pole Test and Rotarod Test

The pole test was performed following a modified protocol previously described [34]. In particular, a 0.5 m long pole was placed vertically in the home cage and the mice were placed high. The time required to re-descend into the cage was recorded. The mice were trained on the pole test for two consecutive days. Each mouse received three consecutive training trials, each separated by 30 min rest periods. On the third day, the mice were tested and the average time to return to the cage was recorded. Furthermore, a score value was calculated based on the performance of the mouse, as previously reported [27].

The acceleration rotarod test was conducted as previously described [5]. Briefly, the mice were placed on the rotarod (Ugo Basile, Varese, Italy) in acceleration, which accelerated from 2 to 20 rpm in 300 s and the latency at the fall was recorded. The time each rodent managed to stay on top of the rotarod was recorded.

### 2.5. ELISA Test

At the end of motility tests, mice were euthanized by cervical dislocation and the blood was processed to quantify the cytokines. Approximately 500 µL of blood from each animal was centrifuged at 1000× *g* for 10 min, and the serum was used for enzyme-linked immunosorbent assay (ELISA) determination of interleukin 1β (IL-1β), interleukin 6 (IL-6) and interleukin 10 (IL-10). These serum samples were tested with ELISA KIT (Thermo Fisher, Monza, Italy) following the manufacturer’s protocol.

### 2.6. Statistical Analysis

All data are expressed as mean ± standard error of the mean (S.E.M.) and analysed by ordinary one-way ANOVA followed by Dunnett’s multiple comparisons test for the comparison of individual means. All the statistical analysis was obtained by using the GraphPad Prism version 6.01 (GraphPad Software, San Diego, CA, USA).

## 3. Results and Discussion

### 3.1. Chemical Components in GP Extracts

From the analysis by UHPLC/Q-TOF-MS of the dry extract of GP, secondary metabo-lites with several potential therapeutic applications have been identified. The most representative molecules were selected and discussed below. GP could have adaptogenic, anti-inflammatory, antidiabetic, lipid-lowering and anti-arthritic effects in patients [21,24,25]. Many of these functions have been associated with the molecules identified in this work.

#### 3.1.1. Saponins

Saponins are glycosides with functional groups of triterpenoids or spirostanols [35]. They are mainly synthesized in higher terrestrial plants and in a few marine organisms. Like all secondary metabolites produced by plants, the saponin content is also influenced by various biotic stimuli such as parasite attacks, pathogenic infections, the mutualistic symbiosis of plants with rhizobium bacteria and mycorrhizal fungi [35]. Saponins are functional components of many plant-based drugs and popular medicines and have shown an important function in improving the inflammatory, immune and anticancer response and in reducing cholesterol [36,37]. Furthermore, saponins showed good activity in cellular protection from ischemia and hypoxia [38]. Based on their structure, saponins are mainly classified into two classes: triterpene saponins and steroid saponins. The saponins identified in the GP samples are of the triterpene type (Table 1). Ginsenosides are the main saponins identified in our GP samples, reaching a total of 7.2 mg/L (as azadirachtin B equivalent). Ginsenosides are predominantly present in *Areliaceae* such as in *Panax ginseng*, and *Gynostemma pentaphyllum* is the only plant not belonging to the Araliaceae that contains ginsenosides [39]. Natural ginsenosides must first be transformed into secondary saponins by the metabolism of the gastrointestinal flora before being absorbed, as observed by He and colleagues [35]. Their main clinical applications concern pathologies with an inflammatory basis, cardiovascular diseases, diabetes and gastrointestinal diseases [40]. The specific triterpene saponin of *Gynostemma pentaphyllum* is gypenoside. Interestingly, we detected both gypenoside XXV and gypenoside XLVI in the GP extracts. Indeed, gypenoside has shown anti-aging, anticancer, hypoglycaemic, lipid-lowering and neuroprotective effects, as reported by some authors [22,41,42,43]. In addition, the environment can influence the synthesis and accumulation of gypenosides. For instance, some fungi present in the soil can stimulate or inhibit the synthesis of gypenosides [39].

#### 3.1.2. Polyphenols

**Figure 3:**
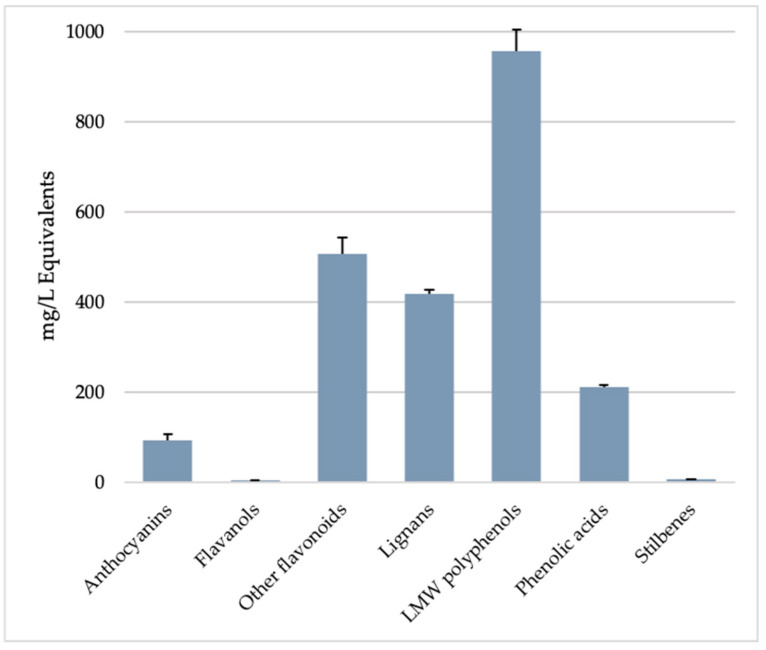
Phenolics composition (as mg/L Equivalents) in *Gynostemma pentaphyllum* (var. Ginpent) extracts. Abbreviation: LMW: Low molecular weight. Semi-quantitative data per class were obtained from cumulate abundances, using pure standards. In more detail, cyanidin (anthocyanins), catechin (flavanols), luteolin (flavones and remaining flavonoids), sesamin (lignans), ferulic acid (hydroxycinnamic acids and other phenolic acids), resveratrol (stilbenes), and tyrosol (low molecular weight phenolics) were used.

To date, nearly 10.000 molecules belonging to the polyphenols class have been identified among the secondary metabolites produced by plants. Apart from the structural functions that phenolic derivatives perform in plants such as lignin, polyphenols are antioxidant molecules with a protective function of photosynthetic systems against excessive radiation. Moreover, they can attract animals to the flowers and fruits of plants [1,2]. In recent decades, polyphenolic compounds have attracted great attention from a pharmaceutical point of view for their antioxidant and anti-inflammatory properties. They have beneficial effects on health, both in animal models and in clinical trials [44,45,46,47]. Figure 3 shows the main classes of polyphenols identified in GP extracts. The class of low molecular weight (LMW) polyphenols was the most abundant (i.e., 956 mg/L eq.), followed by other flavonoids (such as flavonols, flavones, flavanones, and chalcones group), lignans, and phenolic acids (507, 418 and 211 mg/L eq., respectively). The polyphenols identified in our GP samples have shown the ability to modulate LPS-mediated inflammation, as reported by several authors [5,10,48]. Cyanidins, peonidins or malvidins and other derivatives have been identified and included in the group anthocyanins, also with *O*-glucosides modification that is important for the stability and activity of these compounds [49]. Anthocyanins are important for the determination of colours in plants and show remarkable antioxidant properties [48,50]. In addition, anthocyanins prevent the action of reactive oxygen species (ROS) generated by the administration of LPS, as observed by some authors [48,51]. Another polyphenol identified and worthy of interest is ferulaldehyde, a hydroxycinnamaldehyde found to be able to reduce the inflammatory response by reducing the release of cytokines, such as interleukin-1β and TNF-α, and by inhibiting the LPS-induced activation of the nuclear factor kappaB pathway in mice models [52]. Interestingly, some authors reported a synergistic effect of ferulaldehyde with Methotrexate in the therapy of inflammatory arthritis [52]. Among other molecules, sinapine is one of the most representative phenolic acids in our samples, followed by *m*-coumaric acid and 2-hydroxybenzoic acid. These compounds have been detected mainly in the methanolic GP extracts and these chemical structures exert strong antioxidant properties [53]. Indeed, sinapine can counter reactive oxygen species (ROS) by interacting with mitochondrial systems, as observed by several authors in cardiomyocyte cultures [54]. Interesting data have been collected on cirsimaritin, a dimethoxy flavone present in some plants. This molecule exhibits potent antimicrobial, antioxidant, and antispasmodic activities and also inhibits cyclooxygenase-1 (COX-1) [55]. Furthermore, it appears to block inflammatory action by inhibiting the expression of interleukin-6 (IL-6) and the activity of tumour necrosis factor-α (TNF-α) in cultures of LPS-stimulated macrophages [56]. Inhibitory actions of the inflammatory process were also observed in animal models treated with dihydroquercetin, isorhamnetin and anethole [57].

#### 3.1.3. Phytosterols

Table 2 shows the main phytosterols identified in our GP samples. Penasterol (4,4-dimethyl-14-carboxy-cholesta-9(10),24-dien-3beta-ol) has been shown to be the most abundant sterol detected in our extracts (as 178.19 mg/L Eq.). This last sterol was first isolated from Okinawan marine sponge *Penares sp.* and was reported to have potent antileukemic activity [58]. Phytosterols are plant sterols located in the cell membrane. Moreover, sitosterol-β-glucoside (3-*O*-(6′-*O*-(7*Z*,10*Z*-hexadecadienoyl)-beta-d-glucopyranosyl)-stigmast-5-en-3beta-ol) was found to be involved as a primer for cellulose synthesis in plants by the action of glucosyltransferase [59]. Phytosterols are also employed in many food supplements used as adjuvants in lipid-lowering therapies; however, some data have also been collected in the inflammatory context. Phytosterols exert a significant topical anti-inflammatory activity in animal models, modulate the T-helper immune response in vivo, and show marked endothelial anti-inflammatory activity [60]. The anti-inflammatory action results in a reduction in the expression of inflammatory cytokines and the synthesis of anti-inflammatory ones [60]. Plant sterol consumption was also regulated by the European Food Safety Agency for health claims. Indeed, it was suggested that 3 g/day plant sterols/stanols can reduce blood LDL-cholesterol and the risk of heart diseases. Furthermore, interesting data have been observed regarding rheumatic pathologies. Indeed, phytosterols have an anti-stiffness action in the therapy of rheumatic diseases in mouse models [61]. Additionally, paw oedema and neutrophils infiltration into inflamed tissues in animal models of induced arthritis are reduced after the administration of phytosterols [61]. On the other hand, in humans it was observed that professional runners who consumed phytosterols showed a reduced production of IL-6 and other inflammatory parameters [60]. The action of phytosterols at the arthritic level is linked to the penetration of phytosterols into the cell membranes of the chondrocytes of the cartilage tissue, where they perform an anti-inflammatory action.

### 3.2. GP Restores Motor Performance after Treatment with LPS

In order to evaluate the effects on the motor performance of animals pre-treated with GP and subjected to an inflammatory stimulus (LPS), the pole test and rotarod test were performed following the experimental design shown in Figure 2.

#### 3.2.1. Pole Test

**Figure 4:**
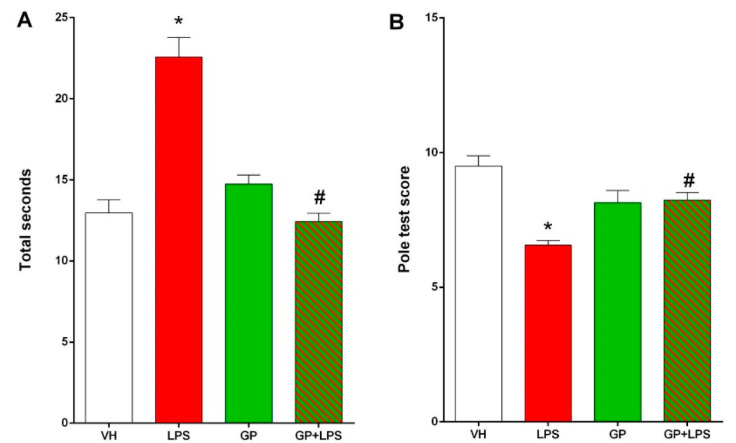
Motor coordination test: pole test. (**A**) Motor performance measured as total seconds taken by the mouse to return to its cage; (**B**) motor performance measured as score. The score is the algebraic sum of the values given to the time taken by the mouse to rotate on itself, to descend to the upper half, to descend to the lower half and to complete the total length of the pole. If the mouse completes every single step within 4 s, 3 points are awarded, within 8 s 2 points are awarded and over 8 s 1 point is awarded. Data are shown as the mean ± SEM, and ordinary one-way ANOVA followed by Dunnett’s multiple comparisons test was used for statistical significance; * *p* < 0.005 vs. VH; # *p* < 0.005 vs. LPS.

The pole test (Figure 4) measures the motor coordination of the animal. The mouse is placed with its head facing upwards on a vertical pole immersed in the animal’s cage. Initially, the operator measures the time taken for the animal to change direction and return to its cage in basal conditions (without treatment) and establishes an average time. On the day of the experiment, the operator measures the time taken for the animal to return to its cage (Figure 4A) and establishes a “score” with respect to the basal time (Figure 4B). High “score” values correspond to performances equal to or higher than those observed in baseline.

Animals treated with LPS show significantly lower performance in motor coordination in the pole test than animals receiving vehicle only (*p* < 0.05). On the contrary, animals pre-treated with GP and subsequently receiving LPS do not show altered performance compared to the control group (VH) (Figure 4). The effects on motor performance after different LPS treatments have been abundantly reported and discussed in the literature [5,27,62,63,64]. LPS at both the cerebral and peripheral levels trigger an inflammatory response that alters motor skills [27,62]. Indeed, the peripheral administration of LPS can trigger an alteration in the coordination of the motor response in the brain [62]. Likewise, the pole test was previously used to assess motor disorders associated with basal ganglia in mice [34]. The coordination of movement is one of the main functions of the basal ganglia of vertebrates, and neuro-inflammation compromises the local neuronal transmission [62]. On the other hand, LPS often generates a local inflammatory response that negatively affects motor coordination [65]. Indeed, LPS induces the release of inflammatory cytokines (see below), which could contribute to the impaired motor coordination observed in our experiments. Pre-treatment with GP appears to protect against motor deficits induced by LPS treatment. The different components identified in the GP extract could together contribute to improving the body’s response to the inflammatory stimulus [66].

#### 3.2.2. Rotarod Test

**Figure 5:**
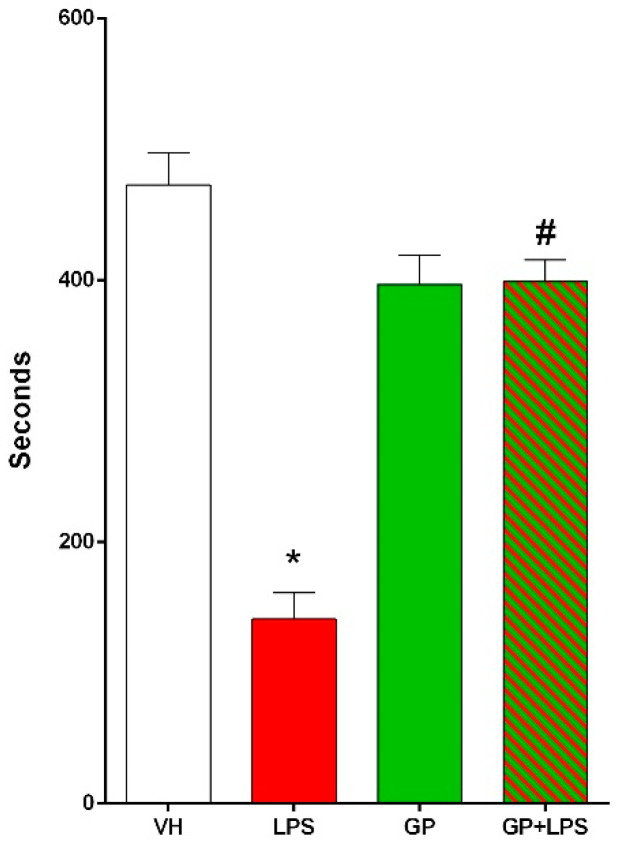
Rotarod test. Time, expressed in seconds, that the animal remains on the moving cylinder. Data are shown as the mean ± SEM, and ordinary one-way ANOVA followed by Dunnett’s multiple comparisons test was used for statistical significance; * *p* < 0.005 vs. VH; # *p* < 0.005 vs. LPS.

Alterations in motor coordination were also observed in the rotarod test (Figure 5). The rotarod test is one of the most commonly used tools to test motor coordination and balance in mice. During the test, the mice must be able to walk on a rotating cylinder subjected to acceleration. The test measures the latency when mice fall from the rotating cylinder. Different mouse models of neurological diseases such as Parkinson’s disorders, amyotrophic lateral sclerosis, cerebellar ataxia, traumatic brain injury and stroke show poor performance on the rotarod test [34]. Furthermore, even the animals treated with LPS show strong difficulties in maintaining balance following the acceleration in the rotarod test [34]. As observed in the pole test, pre-administration of GP protects against LPS-induced motor coordination alteration in the rotarod test. The data graphically reported in Figure 5 show that animals treated with LPS spend significantly less time than the control group on the rotating cylinder. On the contrary, animals that received GP and LPS spent significantly more time on the rotating cylinder compared to the LPS group. Indeed, more significant motor coordination skills have been observed in animals pre-treated with GP and subsequently with LPS than the LPS group. Since GP has no effect (ameliorative or pejorative) on the motor coordination of animals that do not receive LPS, its action, in our mouse model, would have a protective function against the inflammatory stimulus only. As discussed above, *Gynostemma* genus contains certain metabolites that enhance muscle proliferation by activating adenosine monophosphate-activated protein kinase (AMPK) signalling pathways [67]. In particular, extracts of *Gynostemma* species restore the ability to move in animal models with some motor alterations [67].

### 3.3. GP Decreases the Inflammatory Cytokines after Treatment with LPS

**Figure 6:**
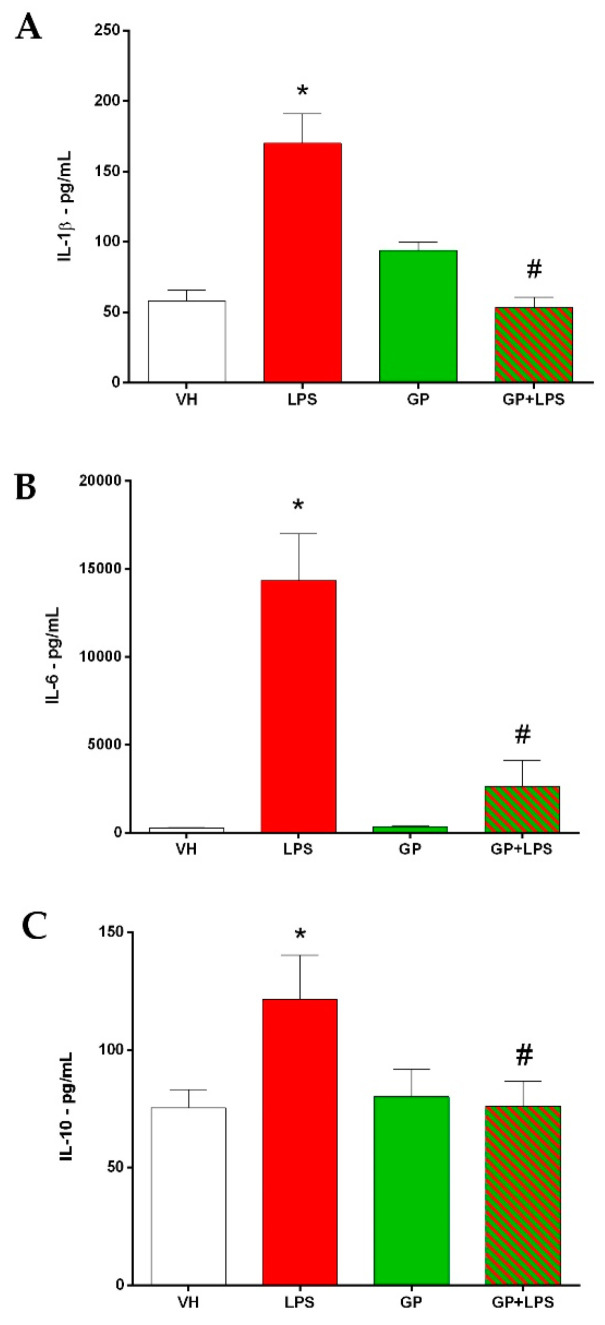
Quantification of interleukin 1β (**A**), interleukin 6 (**B**) and interleukin 10 (**C**) from mice serum. Data are shown as the mean ± SEM, and ordinary one-way ANOVA followed by Dunnett’s multiple comparisons test was used for statistical significance; * *p* < 0.005 vs. VH; # *p* < 0.005 vs. LPS.

Inflammatory cytokines are molecular messengers released by the body in response to an inflammatory stimulus (bacteria, viruses and parasites) [68]. These signals act at the site of the inflammatory stimulus but can also induce a systemic response. In this work, the release of three cytokines involved in inflammation were measured: interleukin-1β (IL-1β), interleukin 6 (IL-6), and interleukin 10 (IL-10). The quantification was performed by means of an ELISA test (enzyme-linked immunosorbent assay) specific for the three cytokines. In Figure 6, it can be seen how the animals treated with LPS show a significant production of IL-1β and IL-6, two cytokines that promote inflammation. Indeed, it is known that the LPS stimulus causes an increase in the expression and release of IL-1β and IL-6, as reported by many authors [5,68,69,70,71]. The inflammatory response also depends on the LPS’ dosage. In our model, the use of 7.5 mg/kg of LPS triggered a considerable inflammatory response. At the same time, the release of IL-10 into the bloodstream of LPS-treated mice also increased. IL-10 is considered an anti-inflammatory cytokine, and its expression correlates with acute inflammatory states as observed by Turner and colleagues [68]. Indeed, the increase in its expression determines, in the later stages of inflammation, a decrease in the release of inflammatory cytokines. Therefore, in our experimental protocol, its increase in animals treated with LPS would indicate a compensatory action of the organism to counteract the inflammatory response. Data obtained demonstrate that GP blocked the LPS-induced cytokine response. Indeed, the animals pre-treated with GP and which subsequently received LPS did not show an increase in the production of IL-1β, IL-6 and IL-10 compared to the control group (VH). Therefore, GP appears to block the inflammatory response by acting on the cytokines. This action can be associated with the multiple components identified in GP extract such as saponins, polyphenols and phytosterols. Indeed, many authors have reported the contribution of molecules belonging to these three classes in reducing inflammatory cytokines [22,23,37,38,48,56,60,67,72]. Furthermore, we hypothesize that the anti-inflammatory action of GP can be attributed to the ability of the entire phytocomplex to block the effects of LPS, as already observed in many other plant species [67,72,73,74].

## 4. Conclusions

The data collected in this work highlighted the ability of GP to prevent both motor alterations and the inflammatory response in LPS-treated mice. LPS is found in the outer cell wall of Gram-negative bacteria and triggers the inflammatory response by binding to specific receptors located in monocytes, dendritic cells, macrophages and B cells, promoting the secretion of pro-inflammatory cytokines, nitric oxide and eicosanoids [68]. This inflammatory reaction can also interfere with motor coordination. Indeed, some nerve circuits are responsible for the central motor coordination that transmits the signalling to the peripheral muscles [62]. The inflammatory reaction triggered by LPS can alter motor coordination both in the brain and in the muscles [5,27,62]. Our results confirmed the inflammatory response and motor alterations in LPS-treated animals. In addition, animals pre-treated with GP and subsequently with LPS did not show alterations in inflammatory signalling and motor coordination. Our data suggested that the protective action might derive from the different molecules present in GP. This extract allows the exploitation of the therapeutic effects of all the components of the GP phytocomplex, such as saponins, polyphenols and phytosterols, identified in this work. In this regard, plant extracts showed remarkable anti-inflammatory properties thanks to the presence of multiple molecules [66,75]. At the same time, other authors highlighted the anti-inflammatory actions of single molecules purified from plant extracts [5,36,38]. Further studies will be needed to evaluate whether the phytocomplex of GP shows a greater anti-inflammatory power than any single molecule present in the same phytocomplex. In addition, ecological aspects of GP (soil nutrients, abiotic and biotic stress, intensity of light radiation, etc.) that can affect the content of secondary metabolites will have to be considered.

In conclusion, GP showed a protective effect against the inflammatory stimulation generated by the LPS. These data strengthen the therapeutic value of GP with important applications both in the human and veterinary field.

## Figures and Tables

**Table 1:** Semi-quantification of selected saponins (as mg/L Equivalent) detected in *Gynostemma pentaphyllum* (var. Ginpent) extracts.

Name	Formula	mg/L ± SD (as Ginsenoside-Rb eq.)
Ginsenoside “RF1”/“RF2”/“RF2-2”	C42 H72 O13	4.67 ± 0.23
Protopanaxatriols	C30 H52 O4	1.66 ± 0.10
Ginsenoside RH2	C36 H62 O8	1.44 ± 0.05
Protopanaxadiols	C30 H52 O3	1.27 ± 0.02
Ginsenoside RD	C48 H82 O18	1.07 ± 0.02
Gypenoside XXV	C47 H78 O18	1.06 ± 0.02
Ginsenoside RF	C42 H72 O14	0.68 ± 0.01
Isofucosterolo	C29 H48 O	0.52 ± 0.01
Ginsenoside RB1	C52 H92 O23	0.36 ± 0.02
Gypenoside XLVI	C48 H82 O19	0.31 ± 0.05
Phyllodulcin	C16 H14 O5	0.09 ± 0.01

Data are presented as mean values ± standard deviation of three independent replicates.

**Table 2:** Semi-quantification of selected phytosterols (as mg/L equivalent) detected in *Gynostemma pentaphyllum* (var. Ginpent) extracts.

Name	Formula	mg/L ± SD (as cholesterol eq.)
4,4-dimethyl-14-carboxy-cholesta-9(10),24-dien-3beta-ol	C30 H48 O3	178.19 ± 14.04
3-*O*-(6′-*O*-(7*Z*,10*Z*-hexadecadienoyl)-beta-d-glucopyranosyl)-stigmast-5-en-3beta-ol	C51 H86 O7	62.35 ± 2.08
(22R)-22-hydroxystigmast-4-en-3-one	C29 H48 O2	53.60 ± 7.39
(22S)-1alpha-acetoxy-5alpha-furospirostan-3alpha,11beta,20*R*-triol	C30 H48 O7	36.04 ± 2.11
3beta-hydroxy-4alpha-methyl-5alpha-cholest-7-ene-4beta-carboxylic acid	C29 H48 O3	32.70 ± 6.02
4-methylene-5alpha-poriferast-8(14)-en-3beta,7alpha,15beta-triol	C30 H50 O3	30.20 ± 0.95
3beta-acetoxy-cholest-5-en-7-one	C29 H46 O3	29.17 ± 5.98
4-methylene-5alpha-poriferast-8(9)-en-3beta,11beta,14alpha,15alpha-tetrol	C30 H50 O4	26.13 ± 1.02
24-isopropenyl-cholesta-5,22*E*-dien-3beta-ol	C30 H48 O	24.38 ± 4.66
Other phytosterols		857.00 ± 49.67

Data are presented as mean values ± standard deviation of three independent replicates.

## Data Availability

The data presented in this study are available on request from the corresponding author.
